# Pterostilbene-Incorporated Tissue Conditioners Exhibit Sustained Antifungal Activity Against *Candida albicans* In Vitro with Preserved Biocompatibility

**DOI:** 10.3390/ma19102126

**Published:** 2026-05-19

**Authors:** Teuta Komoni, Ivana Sutej

**Affiliations:** 1 Faculty of Dentistry, University for Business and Technology-UBT, 10000 Prishtina, Kosovo; teuta.komoni@ubt-uni.net; 2 School of Dental Medicine, University of Zagreb, 10000 Zagreb, Croatia

**Keywords:** pterostilbene, tissue conditioner, functional biomaterial, antifungal, *Candida albicans*, drug delivery, cytocompatibility

## Abstract

*Candida albicans*-associated denture stomatitis is a common inflammatory condition in denture wearers. Conventional tissue conditioners provide temporary relief but lack intrinsic antifungal activity, allowing persistent microbial colonization and biofilm formation. Functionalization with bioactive agents represents a promising preventive strategy. This study evaluated the antifungal efficacy and biocompatibility of pterostilbene (PTE), a natural stilbenoid compound, incorporated into a commercially available tissue conditioner. Antifungal activity of PTE against *C. albicans* ATCC 10231 was evaluated using broth microdilution and XTT biofilm assays. Tissue conditioner discs containing 1% and 2.5% (*w*/*w*) PTE were fabricated and tested after 24 h, 72 h, and 1 week using colony-forming unit (CFU) counts and metabolic activity assays. Biocompatibility was assessed by exposing mouse embryonic fibroblast (MEF) cells to conditioned eluates followed by an MTT viability assay. PTE inhibited biofilm formation in a concentration-dependent manner, with significant suppression observed at ≥8 µg/mL (*p* < 0.001). A time-dependent antifungal effect was observed over one week. PTE-functionalized tissue conditioners significantly reduced fungal adhesion compared with controls at all-time points (*p* < 0.001). Cell viability remained above 70%, meeting ISO 10993-5 criteria for non-cytotoxicity, indicating potential for localized prevention of denture stomatitis.

## 1. Introduction

Denture stomatitis is a prevalent inflammatory condition among denture wearers and is strongly associated with colonization of denture surfaces by *Candida albicans* [1,2]. Although *C. albicans* is a commensal microorganism of the oral cavity, it may become pathogenic under favorable local or systemic conditions, leading to opportunistic infections and persistent mucosal inflammation [3]. Tissue conditioners are widely used during the management of denture stomatitis and the correction of denture-related issues. These temporary soft lining materials redistribute occlusal forces, reduce mechanical irritation, relieve pain, and promote mucosal healing [4]. However, tissue conditioners present inherent disadvantages, including surface roughness, porosity, and plasticizer leaching, which facilitate microbial adhesion and biofilm formation [5]. Consequently, tissue conditioners serve as favorable substrates for *C. albicans* adhesion, colonization, and persistence, largely due to their porous and plasticized structure, which facilitates microbial adhesion and biofilm formation [6,7]. Therefore, transforming these materials from passive liners into bioactive therapeutic platforms represents a promising strategy for controlling microbial colonization at the denture–mucosa interface [8,9,10,11,12]. The management of oral candidiasis primarily relies on antifungal agents belonging to the polyene, fluoropyrimidine, and azole classes [13]. Despite their clinical effectiveness, prolonged or repeated use of these agents has been associated with adverse effects and the emergence of antifungal resistance [14]. Increasing resistance among oral *Candida* isolates has been reported, posing a significant challenge to long-term treatment success [15]. These limitations have stimulated growing interest in alternative or adjunctive antifungal strategies, particularly those based on naturally derived bioactive compounds [16]. Pterostilbene is a naturally occurring stilbenoid structurally related to resveratrol and predominantly found in *Vaccinium species* [17]. Compared to resveratrol, pterostilbene exhibits enhanced lipophilicity, improved bioavailability, and greater metabolic stability, making it particularly suitable for integration into polymer-based dental materials requiring sustained bioactivity [18]. In addition to its well-documented antioxidant and anti-inflammatory properties, pterostilbene has demonstrated promising antimicrobial activity [19,20]. Previous studies have shown that pterostilbene effectively inhibits microbial growth and biofilm formation, including significant antifungal activity against *C. albicans* [21,22,23]. However, despite its demonstrated efficacy, data regarding its use as a functional additive in dental materials—particularly tissue conditioners—and its potential effects on antifungal performance and biocompatibility remain limited [24,25,26]. Moreover, material-based antifungal strategies have been explored in prosthodontics, focusing on the functionalization of tissue conditioners with antifungal agents or bioactive compounds to prevent fungal colonization on denture surfaces. Previous studies have shown that functionalization of tissue conditioners with natural compounds, essential oils, or nanoparticles significantly reduces *C. albicans* adhesion and growth while maintaining acceptable mechanical properties [8,9,10,11,12,26,27]. For example, incorporation of plant-derived extracts such as Cnidium officinale has demonstrated antifungal efficacy with acceptable biocompatibility [26]. Essential oils including Origanum, Melaleuca, and Ocimum basilicum have also shown antifungal activity when incorporated into tissue conditioners [8,10,11], while, nanoparticle-based approaches such as ZnO–Ag systems have demonstrated enhanced antimicrobial performance [9]. More recently, antimicrobial-loaded systems have further explored strategies to achieve sustained antimicrobial effects within tissue conditioners [28]. However, these approaches are predominantly evaluated using diffusion-based or inhibition assays, which assess antifungal activity indirectly and do not fully reflect *C. albicans* behavior on the material surface. In addition, although colony-forming unit–based methods are also used to quantify viable microorganisms, these measurements alone do not capture metabolic activity or provide a comprehensive assessment of biofilm viability and function. As a result, these approaches provide limited insight into biofilm persistence and functional viability on tissue conditioners. In addition, natural extracts represent chemically complex and variable mixtures, which may lead to inconsistencies in bioactivity and reproducibility, whereas nanoparticle-based systems may introduce concerns related to cytotoxicity and potential alterations in material properties [28,29,30]. Importantly, biocompatibility is often not comprehensively evaluated using relevant cell-based models under conditions reflecting material eluate exposure over time. Specifically, no studies to date have evaluated the incorporation of pterostilbene into tissue conditioners with simultaneous assessment of antifungal efficacy and biocompatibility under direct-contact conditions.

In contrast, the present study evaluates antifungal and antibiofilm activity under direct-contact conditions, focusing on the persistence of *C. albicans* on the material surface over time, while simultaneously assessing biocompatibility using a fibroblast model. Furthermore, the use of pterostilbene as a chemically defined compound offers improved stability and reproducibility compared to complex extracts and avoids limitations associated with nanoparticle-based systems.

Therefore, we hypothesized that the incorporation of pterostilbene into a tissue conditioner would confer sustained antifungal and antibiofilm activity against *C. albicans* without compromising material biocompatibility. This study seeks to provide experimental evidence supporting the potential use of pterostilbene-functionalized tissue conditioners as a novel prosthodontic approach for the prevention and management of *C. albicans*-associated denture stomatitis.

**Figure 1:**
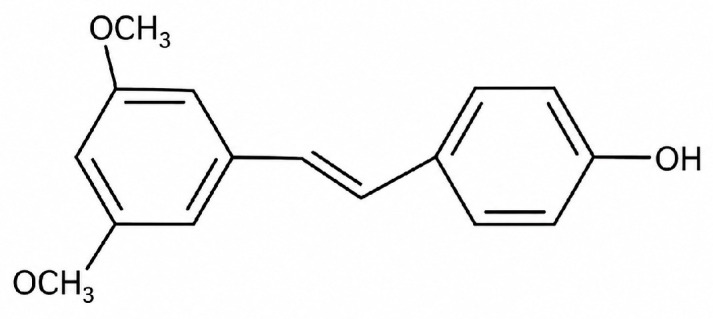
Chemical structure of pterostilbene (trans-3,5-dimethoxy-4′-hydroxystilbene).

## 2. Materials and Methods

### 2.1. Study Design

This experimental in vitro study was conducted in three sequential stages to evaluate the antifungal, antibiofilm, and biocompatibility properties of pterostilbene (PTE) as a functional agent when incorporated into a commercially available tissue conditioner (TC). The study was designed in accordance with previously published protocols for antifungal susceptibility testing, biofilm quantification, tissue conditioner functionalization, and cell biocompatibility assays.

### 2.2. Chemicals and Reagents

Pterostilbene (PTE, ≥98% purity, Cayman Chemical, Ann Arbor, MI, USA) was used as the candidate bioactive functional compound (Figure 1). Ketoconazole (Cayman Chemical, USA) served as the positive antifungal control. Dimethyl sulfoxide (DMSO) was used as a solvent vehicle where required. RPMI 1640 medium buffered with MOPS supplemented with L-glutamine, Sigma-Aldrich (Darmstadt, Germany), was used for antifungal susceptibility testing, while Sabouraud Dextrose Agar (SDA) was used for fungal culturing. XTT sodium salt (Cayman Chemical, USA) and menadione, Sigma-Aldrich (Germany), were used for metabolic activity assays. The final concentration of DMSO did not exceed 1% (*v*/*v*).

### 2.3. Microorganism and Culture Conditions

A standard reference strain of *Candida albicans* ATCC 10231 was used throughout the study to ensure reproducibility and reduce variability related to strain-specific resistance. Yeast cultures were maintained on SDA plates and subcultured in RPMI 1640 at 37 °C prior to experimentation. A single colony was suspended in phosphate-buffered saline (PBS, pH 7.4) and adjusted to a 0.5 McFarland standard (1–5 × 10^6^ CFU/mL). A working suspension of 1–5 × 10^5^ CFU/mL was prepared by serial dilution in RPMI-1640 medium.

### 2.4. Antifungal and Antibiofilm Screening

Initial screening involved testing PTE and positive control Ketoconazole for antifungal activity against *C. albicans*. Broth microdilution assays were performed according to EUCAST (European Committee on Antimicrobial Susceptibility Testing) and CLSI M27-A3 (Clinical and Laboratory Standards Institute) guidelines [31,32]. Biofilm formation was induced in sterile, flat-bottom 96-well microtiter plates by inoculating 100 µL of standardized yeast suspension per well. Plates were incubated statically at 37 °C for 24 h. Growth controls (drug-free medium with inoculum) and sterility controls (medium only) were included. PTE and Ketoconazole concentrations ranging from 4 to 64 µg/mL were tested. Biofilm formation was evaluated using visual scoring. Visual scoring was performed using a semi-quantitative scale (0–3), where 0 = no visible biofilm, 1 = weak, 2 = moderate, and 3 = strong biofilm formation, based on turbidity and surface adherence after washing. Mature biofilms were washed, mechanically disrupted, serially diluted, and plated onto SDA. After incubation at 37 °C for 24 h, colonies were counted and expressed as CFU/mL. XTT assays were performed. Biofilm metabolic activity was assessed using the XTT reduction assay. Pre-washed biofilms were incubated with XTT reagent for 1 h at 37 °C, and absorbance was measured at 490 nm. Raw absorbance values were background-corrected (blank subtraction) and normalized to untreated controls (set as 100%) to allow comparison across conditions. Biofilm metabolic activity (%) was calculated directly from normalized XTT absorbance values relative to untreated controls (set as 100%) and represents experimentally measured biofilm metabolic activity. MIC was defined as the lowest concentration that met the EUCAST/CLSI broth microdilution endpoint after 24 h. IC_50_ and IC_90_ values were calculated using nonlinear regression (four-parameter logistic model) based on normalized XTT data, representing the concentrations required to achieve 50% and 90% reduction in metabolic activity, respectively.

### 2.5. Preparation of PTE-Modified Tissue Conditioner

**Figure 2:**
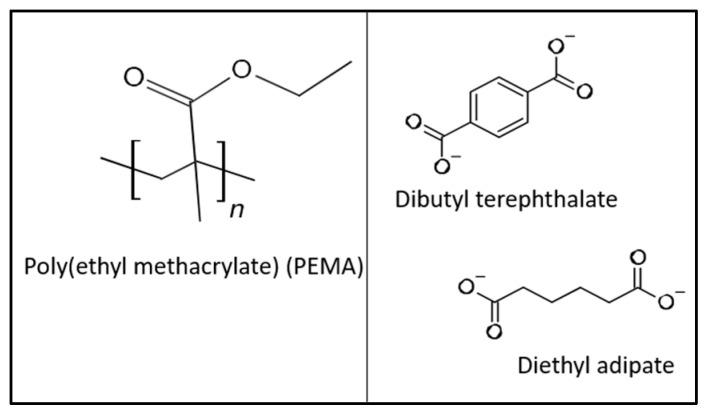
Representative chemical structures of the tissue conditioner matrix, including the poly(ethyl methacrylate) (PEMA) repeating unit and representative plasticizers (terephthalate- and adipate-based compounds).

Pterostilbene was incorporated into a commercially available tissue conditioner (GC Tissue conditioner, GC Europe) to create a functional composite material (Figure 2). A powder-to-liquid mixing ratio of 1.22:1 was used according to manufacturer instructions [7]. PTE was added to the liquid component to obtain a final concentration of 1% (*w*/*w*) and 2.5% (*w*/*w*). Ketoconazole at 1% served as a positive control, while the PTE vehicle solution served as a negative control. Disc specimens (8 mm diameter, 5 mm thickness) were prepared and allowed to set.

### 2.6. Antifungal Testing of Functionalized Tissue Conditioner Discs

Prepared discs were incubated with *C. albicans* suspensions in 1.5 mL RPMI 1640 medium for 24 h, 72 h and 1 week at 37 °C. Following incubation, discs were vortexed and sonicated in 5 mL phosphate-buffered saline (PBS) to detach adherent biofilms [31,32]. Serial dilutions (1:10 and 1:100) were plated on SDA for CFU determination. XTT reduction assays were additionally performed on recovered suspensions to evaluate the metabolic activity and viability of *C. albicans* cells following exposure to the pterostilbene-modified tissue conditioner. XTT values were background-corrected and normalized to the untreated control (set as 100%) to ensure comparability across time points.

### 2.7. Cytotoxicity/Biocompatibility Testing

For biocompatibility assessment, TC discs containing PTE at 1% and 2.5% were prepared. Control groups included TC without additives and TC containing DMSO. After UV sterilization, discs were incubated in 24-well plates with culture medium. Conditioned media were collected at predetermined time points and stored at −80 °C for further analysis [28,29].

#### Cell Viability Assay

Cell lines were cultured under standard conditions and exposed to the collected conditioned media. Mouse embryonic fibroblast (MEF) cells obtained from ATCC (Manassas, VA, USA) were cultured in Dulbecco’s Modified Eagle Medium (DMEM) supplemented with 10% fetal bovine serum and 1% penicillin/streptomycin. Cells were maintained under humidified conditions at 37 °C with 5% CO_2_. Cell viability was assessed using the MTT assay [30]. Cells were exposed to collected eluates for corresponding time points (24 h and 48 h). MTT reagent was added and incubated for 4 h, followed by solubilization and spectrophotometric measurement at 580 nm (reference 650 nm). Cell viability was expressed as a percentage relative to untreated control cells.

### 2.8. Statistical Analysis

All statistical analyses were performed using GraphPad Prism 8.0 (GraphPad Software, Boston, MS, USA). All experiments were performed in three independent experiments conducted in technical triplicate. Data were expressed as mean ± standard deviation. Data distribution was assessed prior to analysis. One-way ANOVA with Tukey’s post hoc test was applied to concentration-dependent and time-dependent antifungal datasets (Section 3.1 and Section 3.2), while predefined pairwise comparisons between selected groups in biocompatibility assays (Section 3.3) were analyzed using unpaired two-tailed *t*-tests. A *p*-value < 0.05 was considered statistically significant. MIC was defined as the lowest compound concentration that prevented visible growth after 24 h under the selected broth microdilution conditions. IC_50_ and IC_90_ were calculated by nonlinear regression (4PL) fitted to normalized dose–response data, with values obtained by interpolation from the fitted curve. IC_50_ and IC_90_ values represent parameters derived from the fitted concentration–response model and were used to describe inhibitory potency.

## 3. Results

### 3.1. Antifungal Activity of Pterostilbene Against Candida albicans

**Figure 3:**
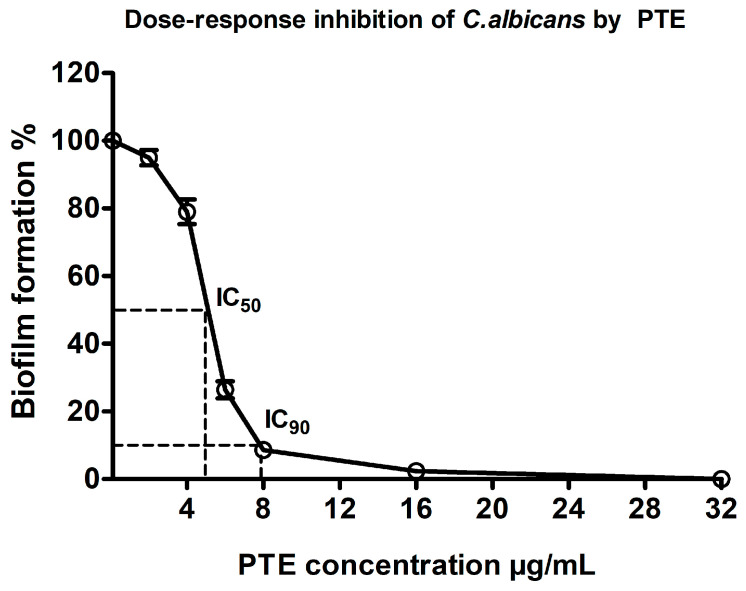
Dose–response inhibition of *C. albicans* biofilm formation by pterostilbene (PTE). Biofilm metabolic activity (%) was calculated from normalized XTT absorbance values relative to the untreated control (set as 100%). Biofilm metabolic activity was quantified using the XTT reduction assay after 24 h incubation. A nonlinear regression (4PL) curve was fitted to normalized dose–response data; IC_50_ and IC_90_ were obtained by interpolation from the fitted curve. Data are presented as mean ± SEM (*n* = 3). Statistical analysis was performed using one-way ANOVA followed by Tukey’s post hoc test.

Pterostilbene (PTE) exhibited a clear concentration-dependent inhibitory effect on *C. albicans* biofilm metabolic activity, as determined by the XTT reduction assay. Increasing PTE concentrations progressively reduced biofilm viability compared with untreated controls (Figure 3). Nonlinear regression (4PL) of the dose–response curve yielded an IC_50_ of 5.1 µg/mL and an IC_90_ of 7.8 µg/mL, obtained by interpolation from the fitted curve. In accordance with MIC-based interpretation of the broth microdilution assay, significant suppression was observed at concentrations ≥ 8 µg/mL compared with the untreated control (*p* < 0.001). These findings demonstrate that PTE possesses potent antifungal and antibiofilm activity against *C. albicans*.

### 3.2. Antifungal Efficacy of PTE-Incorporated Tissue Conditioner

**Figure 4:**
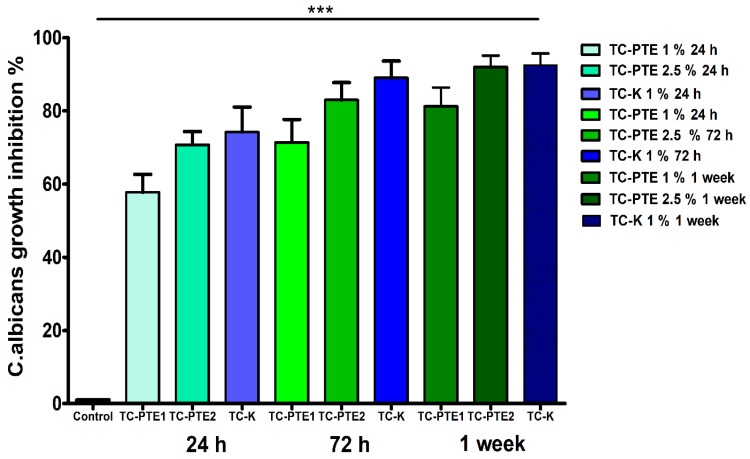
Time-dependent antifungal activity of PTE-modified tissue conditioner discs against *C. albicans*. Colony-forming units (CFU/mL) recovered from tissue conditioner discs containing 1% and 2.5% (*w*/*w*) PTE after 24 h, 72 h, and 1 week incubation. Both formulations significantly reduced fungal adhesion compared with the unmodified tissue conditioner and vehicle control. Sustained inhibition over time supports a time-dependent antifungal effect and the potential of the material as an antifungal biomaterial. Data are expressed as mean ± SEM (*n* = 3). Statistical comparisons were performed using one-way ANOVA with Tukey’s post hoc analysis. *** *p* < 0.001 vs. control.

Tissue conditioner discs functionalized with PTE significantly reduced *C. albicans* adhesion and growth compared with unmodified TC and vehicle control groups at all evaluated time points (Figure 4). Both PTE concentrations (1% and 2.5% *w*/*w*) exhibited measurable antifungal activity after 24 h, which was maintained and slightly enhanced after 72 h and 1 week of incubation (*p* < 0.001). Importantly, the 2.5% PTE formulation consistently demonstrated greater antifungal efficacy than the 1% formulation across all time points, indicating a concentration-dependent activity. This difference reflects a moderate but consistent increase in inhibitory effect for the higher concentration. Both formulations showed a time-dependent increase in antifungal activity over the incubation period.

### 3.3. Biocompatibility of PTE-Modified Tissue Conditioner

**Figure 5:**
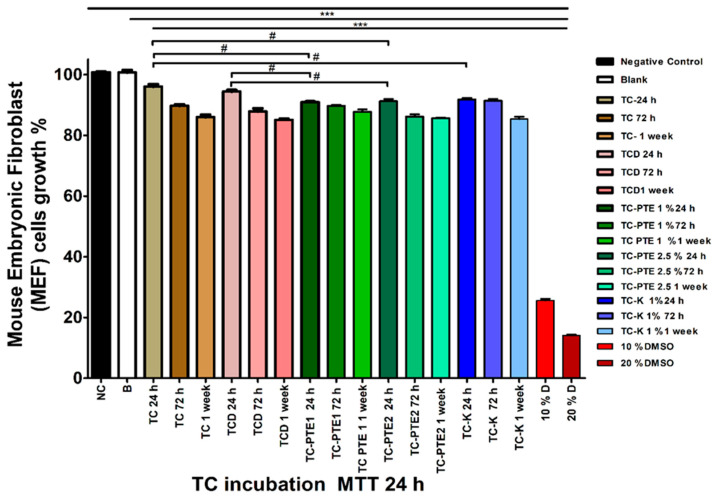
Biocompatibility of PTE-modified tissue conditioner eluates in MEF cells (24 h dataset). Cell viability (%) was assessed by MTT assay after exposure to eluates collected from tissue conditioner materials incubated for 24 h, 72 h, and 1 week. PTE-modified formulations (1% and 2.5%) maintained viability comparable to TC and vehicle control groups. Data are presented as mean ± SEM (*n* = 3). Statistical comparisons between groups at each time point were performed using unpaired two-tailed *t*-tests. *** *p* < 0.001 vs. negative control (NC); # *p* < 0.05 indicates significant differences between groups as indicated by brackets. Within-group comparisons are shown in Table S2; between-group comparisons are provided in Table S3 (including Δ values and exact *p*-values). Groups: NC, negative control; B, blank; TC, tissue conditioner; TCD, vehicle control; TC-PTE 1% and 2.5%, pterostilbene-modified formulations; TC-K, ketoconazole; D, DMSO.

**Figure 6:**
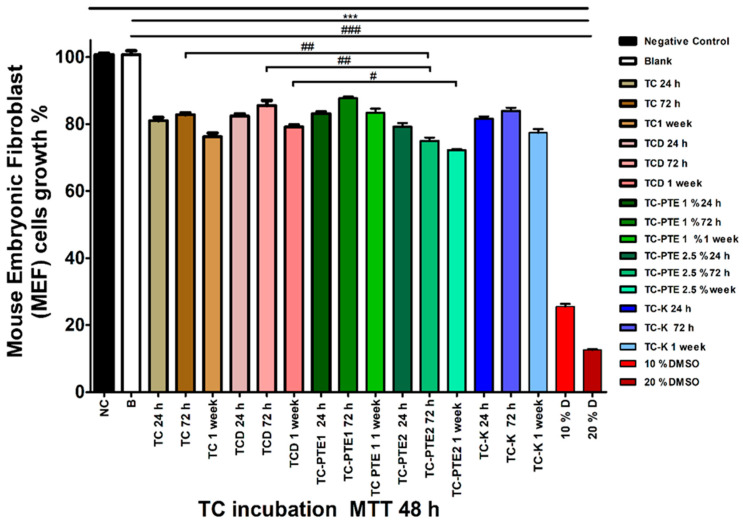
Biocompatibility of PTE-modified tissue conditioner eluates in MEF cells (48 h dataset). Cell viability (%) was assessed by MTT assay after exposure to eluates derived from tissue conditioner materials incubated for 24 h, 72 h, and 1 week. PTE-modified formulations (1% and 2.5%) maintained cell viability within acceptable limits across all conditions. Data are presented as mean ± SEM (*n* = 3). Statistical comparisons between groups at each time point were performed using unpaired two-tailed *t*-tests. *** *p* < 0.001 vs. negative control (NC); # *p* < 0.05, ## *p* < 0.01, ### *p* < 0.001 indicate significant differences between groups as indicated by brackets. Within-group comparisons are detailed in Table S5; between-group comparisons are provided in Table S6 (including Δ values and exact *p*-values). Groups: NC, negative control; B, blank; TC, tissue conditioner; TCD, vehicle control; TC-PTE 1% and 2.5%, pterostilbene-modified formulations; TC-K, ketoconazole; D, DMSO.

The cytotoxicity of eluates obtained from tissue conditioner discs after 24 h, 72 h, and 1 week of material incubation was evaluated using the MTT assay following either 24 h or 48 h exposure of mouse embryonic fibroblast (MEF) cells to conditioned media. Cell viability was expressed as a percentage relative to untreated negative control cells (NC = 100.67 ± 0.45%). Positive controls containing 10% and 20% DMSO produced substantial reductions in cell viability (≥74.5%), confirming the sensitivity of the assay. Across all experimental groups, eluates caused moderate reductions in cell viability compared with NC; however, all values remained above the 70% threshold defined by ISO 10993-5 for non-cytotoxic materials. For eluates collected after 24 h of material incubation, cell viability ranged between approximately 90% and 96% across all formulations, indicating minimal cytotoxic effects (Table S1, Figure 5). With increasing incubation time, modest reductions in viability were observed, particularly for TC-PTE2, although the values remained within acceptable biological limits. Time-dependent analysis showed that TC and TCD groups exhibited gradual increases in cytotoxicity with prolonged incubation, whereas TC-PTE1 maintained relatively stable viability values across all time points. In contrast, TC-PTE2 displayed a progressive increase in cytotoxicity between 24 h and 1 week of incubation, while TC-K showed delayed increases primarily after prolonged incubation (Tables S2, Figure 5). Between-group comparisons revealed that modified formulations occasionally produced slightly greater reductions in cell viability than TC alone during early incubation periods. However, these differences were generally small and diminished after longer incubation times (Table S3, Figure 5). For eluates evaluated following 48 h cell exposure, viability values ranged between approximately 72% and 88% depending on formulation and incubation period (Table S4, Figure 6). TC-PTE2 consistently exhibited the greatest reductions in cell viability, particularly after 72 h and 1 week of material incubation, whereas TC-PTE1 maintained comparatively higher viability values. Detailed statistical comparisons of within-group time-dependent changes and between-group differences are presented in Tables S5 and S6 (Figure 6). Overall, despite modest time-dependent reductions in viability, all tested formulations maintained cell viability above the ISO cytotoxicity threshold, indicating that incorporation of pterostilbene did not compromise the cytocompatibility of the tissue conditioner. Among the tested formulations, the 1% PTE-modified tissue conditioner demonstrated the most favorable balance between antifungal efficacy and biological safety.

## 4. Discussion

In the present study, pterostilbene (PTE) demonstrated significant concentration-dependent inhibition of *C. albicans* biofilm formation. This concentration-dependent reduction in metabolic activity indicates impaired biofilm viability and maturation, providing the basis for hypothesizing a potential antifungal mechanism. These findings are consistent with previous reports, suggesting that the observed effect may involve interference with ergosterol biosynthesis, suppression of hyphal transition, reduction in cell surface hydrophobicity, and modulation of Ras/cAMP signaling pathways involved in biofilm maturation [21,22,23,33]. However, these mechanisms were not directly investigated in the present study and are therefore presented as plausible interpretations supported by the literature. Importantly, antifungal activity was preserved following incorporation into the tissue conditioner matrix. This observation confirms that PTE retains its biological activity within the polymeric environment of the tissue conditioner, supporting its suitability as a functional bioactive additive for dental biomaterials. For comparison, TC discs containing 1% ketoconazole (TC-K) were used as a positive control. TC-K exhibited rapid inhibition of *C. albicans*, reflecting ketoconazole’s strong antifungal activity, whereas the PTE formulations showed more gradual inhibition consistent with a time-dependent antifungal effect. While this pattern may suggest gradual release from the matrix, release kinetics were not directly evaluated in the present study, and this interpretation should be considered preliminary. Notably, the cytotoxicity profile of TC-K was similar to that of the PTE-TC groups, indicating that PTE incorporation did not introduce additional toxicity beyond that of a standard antifungal agent.

From a biomaterial’s standpoint, the most significant observation is the sustained antifungal efficacy of PTE-functionalized tissue conditioners over 1 week. Both 1% and 2.5% formulations significantly reduced fungal adhesion at 24 h, 72 h, and 1 week, indicating a time-dependent antifungal effect. Although this trend is compatible with sustained activity, no release kinetics were assessed. Similar behavior has been reported in polymer-based dental biomaterials [34,35], although this was not directly evaluated in the present study. Such sustained activity is advantageous for preventing initial microbial colonization and biofilm maturation and represents a desirable feature of functional antimicrobial biomaterials, consistent with observations reported in other antimicrobial-modified dental materials [8,9,11,26,28,29,30,36].

Biofilm formation by *C. albicans* is a critical factor in the pathogenesis of denture stomatitis because established biofilms exhibit increased resistance to antifungal agents and mechanical removal. Therefore, strategies aimed at inhibiting early fungal adhesion and biofilm development on denture materials are particularly important for preventing disease progression. By incorporating PTE directly into the tissue conditioner, the material itself becomes an active antifungal surface capable of interfering with fungal colonization [37,38]. Biocompatibility is critical for intraoral application. In this study, eluates from PTE-functionalized tissue conditioners maintained cell viability above 70% under all conditions. According to ISO 10993-5 guidelines, materials exhibiting ≥70% cell viability are classified as non-cytotoxic. Although moderate reductions were observed in some 48 h exposure groups (maximum reduction approximately 28%), values remained within acceptable biological limits. These findings indicate that the concentrations of PTE incorporated into the tissue conditioner did not exert harmful effects on mammalian cells. Notably, the 1% PTE formulation demonstrated the most favorable balance between antifungal efficacy and biocompatibility [26,29,30]. These findings support previous evidence that PTE possesses a favorable safety profile in mammalian systems, including in vivo models demonstrating low toxicity while enhancing survival in experimental candidiasis [23]. Taken together, these results suggest that PTE incorporation does not compromise the biological compatibility of the material, which is an essential requirement for clinical dental biomaterials.

Various antimicrobial strategies have been explored for soft denture liners, including essential oils, plant extracts, silver nanoparticles, and chitosan composites [10,12,19,21,26,27,30,36]. While these approaches often demonstrate strong initial antifungal activity, sustained release and long-term biocompatibility remain important challenges. In comparison, the present findings indicate that PTE incorporation provides moderate yet sustained antifungal activity while maintaining acceptable cytocompatibility, highlighting a favorable balance between antimicrobial effectiveness and biological safety. Localized antifungal delivery via functionalized denture materials offers advantages over systemic antifungal therapy, including reduced systemic exposure, improved patient compliance, and potential reduction in resistance development [37,38]. By targeting fungal adhesion directly at the material surface, PTE-functionalized tissue conditioners may function not only as therapeutic materials but also as preventive biomaterials for denture-associated fungal infections.

Several limitations should be acknowledged. Only a single reference strain (standard laboratory) of *C. albicans* was evaluated, which limits translational relevance, as clinical isolates demonstrate greater genetic and phenotypic heterogeneity, including variability in antifungal resistance and biofilm formation. Inclusion of clinical isolates and mixed-species biofilms would better simulate oral conditions. Furthermore, the present study did not investigate the release kinetics of PTE from the tissue conditioner matrix. Quantitative drug-release analysis and surface characterization would provide further insight into the functional behavior of the modified material. Future investigations incorporating spectrophotometric release profiling, diffusion modeling, and simulated oral environment testing are therefore warranted to better understand the long-term performance of PTE-functionalized tissue conditioners.

## 5. Conclusions

Pterostilbene-incorporated tissue conditioners effectively transform a passive liner into a functional antifungal biomaterial, demonstrating significant in vitro activity against *Candida albicans* while maintaining biocompatibility. This localized approach represents a promising preventive strategy for reducing fungal colonization and the risk of denture stomatitis. A time-dependent antifungal effect was observed, which may indicate sustained activity; however, release kinetics were not directly evaluated and remain to be confirmed. Further studies investigating the release kinetics of PTE and its efficacy in vivo models are required to validate its long-term clinical potential.

## Data Availability

The original contributions presented in the study are included in the article, further inquiries can be directed to the corresponding author.

## Supplementary Materials

The following supporting information can be downloaded at: https://www.mdpi.com/article/10.3390/ma19102126/s1, Table S1. MEF cell viability after exposure to 24 h eluates from tissue conditioner discs. Table S2. Within-group time-dependent cytotoxicity comparisons for 24 h exposure. Table S3. Between-group cytotoxicity comparisons for 24 h exposure. Table S4. MEF cell viability after exposure to 48 h eluates. Table S5. Within-group time-dependent cytotoxicity comparisons for 48 h exposure. Table S6. Between-group cytotoxicity comparisons for 48 h exposure.

## Abbreviations

The following abbreviations are used in this manuscript:

PTE	Pterostilbene
TC	Tissue Conditioner
TC-PTE1	Tissue Conditioner containing 1% Pterostilbene
TC-PTE2	Tissue Conditioner containing 2.5% Pterostilbene
TC-K	Tissue Conditioner containing Ketoconazole
TCD	Tissue Conditioner with Delivery System (Vehicle Control)
CFU	Colony-Forming Unit
MIC	Minimum Inhibitory Concentration
IC50	Half Maximal Inhibitory Concentration
IC90	90% Inhibitory Concentration
MEF	Mouse Embryonic Fibroblast
XTT	2,3-Bis(2-methoxy-4-nitro-5-sulfophenyl)-2H-tetrazolium-5-carboxanilide
DMSO	Dimethyl Sulfoxide
PBS	Phosphate-Buffered Saline
RPMI	Roswell Park Memorial Institute Medium
SDA	Sabouraud Dextrose Agar
ANOVA	Analysis of Variance
SEM	Standard Error of the Mean

## References

[1] Salerno C., Pascale M., Contaldo M., Esposito V., Busciolano M., Milillo L., Guida A., Petruzzi M., Serpico R. (2011). Candida-Associated Denture Stomatitis. Med. Oral Patol. Oral Cir. Bucal.

[2] McReynolds D.E., Moorthy A., Moneley J.O.C., Jabra-Rizk M.A., Sultan A.S. (2023). Denture Stomatitis—An Interdisciplinary Clinical Review. J. Prosthodont..

[3] Cannon R.D., Chaffin W.L. (1999). Oral Colonization by *Candida albicans*. Crit. Rev. Oral Biol. Med..

[4] Mccord J.F., Grant A.A. (2000). Identification of Complete Denture Problems: A Summary. Br. Dent. J..

[5] Anusha (2025). Tissue Conditioners in Prosthodontics—A Narrative Review. https://scope-journal.com/published_paper/1379/Tissue+Conditioners+in+Prosthodontics+-+A+Narrative+Review.

[6] Skupien J.A., Valentini F., Boscato N., Pereira-Cenci T. (2013). Prevention and Treatment of Candida Colonization on Denture Liners: A Systematic Review. J. Prosthet. Dent..

[7] Murata H., Hamada T., Djulaeha E., Nikawa H. (1998). Rheology of Tissue Conditioners. J. Prosthet. Dent..

[8] Srivatstava A., Ginjupalli K., Perampalli N.U., Bhat N., Ballal M. (2013). Evaluation of the Properties of a Tissue Conditioner Containing Origanum Oil as an Antifungal Additive. J. Prosthet. Dent..

[9] Mousavi S.A., Ghotaslou R., Akbarzadeh A., Azima N., Aeinfar A., Khorramdel A. (2019). Evaluation of Antibacterial and Antifungal Properties of a Tissue Conditioner Used in Complete Dentures after Incorporation of ZnO-Ag Nanoparticles. J. Dent. Res. Dent. Clin. Dent. Prospect..

[10] Sharma S., Hegde V. (2014). Comparative Evaluation of Antifungal Activity of Melaleuca Oil and Fluconazole When Incorporated in Tissue Conditioner: An in Vitro Study. J. Prosthodont..

[11] Rajali A., Zain N.M., Amran N.A., Azmi N.H.E. (2023). Antifungal Efficacy of Ocimum Basilicum Essential Oil in Tissue Conditioner Against *Candida albicans*: An In Vitro Study. Contemp. Clin. Dent..

[12] Kula P., Chladek G., Barszczewska-Rybarek I. (2025). Plant-Derived Modifiers for Antimicrobial Soft Denture Liners: A Review. Int. J. Mol. Sci..

[13] Lewis R.E. (2011). Current Concepts in Antifungal Pharmacology. Mayo Clin. Proc..

[14] Niimi M., Firth N.A., Cannon R.D. (2010). Antifungal Drug Resistance of Oral Fungi. Odontology.

[15] Zida A., Bamba S., Yacouba A., Ouedraogo-Traore R., Guiguemdé R.T. (2017). Substances Naturelles Actives Sur *Candida albicans*, Sources de Nouveaux Médicaments Antifongiques: Revue de La Littérature. J. Mycol. Med..

[16] de Oliveira Santos G.C., Vasconcelos C.C., Lopes A.J., de Sousa Cartágenes M.D.S., Filho A.K., do Nascimento F.R., Ramos R.M., Pires E.R., de Andrade M.S., Rocha F.M. (2018). Candida Infections and Therapeutic Strategies: Mechanisms of Action for Traditional and Alternative Agents. Front. Microbiol..

[17] Chan E.W.C., Wong C.W., Tan Y.H., Foo J.P.Y., Wong S.K., Chan H.T. (2019). Resveratrol and Pterostilbene: A Comparative Overview of Their Chemistry, Biosynthesis, Plant Sources and Pharmacological Properties. J. Appl. Pharm. Sci..

[18] Liu Y., You Y., Lu J., Chen X., Yang Z. (2020). Recent Advances in Synthesis, Bioactivity, and Pharmacokinetics of Pterostilbene, an Important Analog of Resveratrol. Molecules.

[19] Lim Y.R.I., Preshaw P.M., Lim L.P., Ong M.M.A., Lin H.S., Tan K.S. (2020). Pterostilbene Complexed with Cyclodextrin Exerts Antimicrobial and Anti-Inflammatory Effects. Sci. Rep..

[20] Mattio L.M., Catinella G., Dallavalle S., Pinto A. (2020). Stilbenoids: A Natural Arsenal against Bacterial Pathogens. Antibiotics.

[21] Hu D.D., Zhang R.L., Zou Y., Zhong H., Zhang E.S., Luo X., Wang Y., Jiang Y.Y. (2017). The Structure-Activity Relationship of Pterostilbene Against *Candida albicans* Biofilms. Molecules.

[22] Simonetti G., Palocci C., Valletta A., Kolesova O., Chronopoulou L., Donati L., Di Nitto A., Brasili E., Tomai P., Gentili A. (2019). Anti-Candida Biofilm Activity of Pterostilbene or Crude Extract from Non-Fermented Grape Pomace Entrapped in Biopolymeric Nanoparticles. Molecules.

[23] Carmo P.H.F.d., Silva M.d.F.d., Fraga A.S., Gonçale J.C., Lima P.M.N.d., Cruz G.M.d., Kemmerich K.K., Ribeiro F.d.C., Garcia M.T., Junqueira J.C. (2025). Antifungal Effects of Pterostilbene on *Candida albicans*, *Candida dubliniensis*, and Microcosm Biofilms of Denture Stomatitis. J. Fungi.

[24] Yudaev P., Chuev V., Klyukin B., Kuskov A., Mezhuev Y., Chistyakov E. (2022). Polymeric Dental Nanomaterials: Antimicrobial Action. Polymers.

[25] Paczkowska-Walendowska M., Kulawik M., Kwiatek J., Bikiaris D., Cielecka-Piontek J. (2025). Novel Applications of Natural Biomaterials in Dentistry—Properties, Uses, and Development Perspectives. Materials.

[26] Lee M.J., Shim Y.S., An S.Y., Kang M.K. (2021). Surface Characterization, Biocompatibility and Antifungal Efficacy of a Denture-Lining Material Containing Cnidium Officinale Extracts. Molecules.

[27] Tas N., Egilmez F., Yiyit Dogan S., Mendi A.H. (2026). Assessment of Mechanical, Physical, and Antimicrobial Properties of Tissue Conditioners Incorporated with Plant Extracts. Odontology.

[28] Naoe T., Hasebe A., Horiuchi R., Makita Y., Okazaki Y., Yasuda K., Matsuo K., Yoshida Y., Tsuga K., Abe Y. (2020). Development of Tissue Conditioner Containing Cetylpyridinium Chloride Montmorillonite as New Antimicrobial Agent: Pilot Study on Antimicrobial Activity and Biocompatibility. J. Prosthodont. Res..

[29] Chaves C.D.A.L., De Souza Costa C.A., Vergani C.E., Chaves De Souza P.P., Machado A.L. (2014). Effects of Soft Denture Liners on L929 Fibroblasts, HaCaT Keratinocytes, and RAW 264.7 Macrophages. BioMed Res. Int..

[30] Kurt A., Erkose-Genc G., Uzun M., Emrence Z., Ustek D., Isik-Ozkol G. (2017). The Antifungal Activity and Cytotoxicity of Silver Containing Denture Base Material. Niger. J. Clin. Pract..

[31] Arendrup M.C., Cuenca-Estrella M., Lass-Flö Rl C., Hope W. (2012). EUCAST Technical Note on the EUCAST Definitive Document EDef 7.2: Method for the Determination of Broth Dilution Minimum Inhibitory Concentrations of Antifungal Agents for Yeasts EDef 7.2 (EUCAST-AFST)*. Clin. Microbiol. Infect..

[32] (2017). Reference Method for Broth Dilution Antifungal Susceptibility Testing of Yeasts.

[33] Li D.D., Zhao L.X., Mylonakis E., Hu G.H., Zou Y., Huang T.K., Yan L., Wang Y., Jiang Y.Y. (2014). In Vitro and in Vivo Activities of Pterostilbene against *Candida albicans* Biofilms. Antimicrob. Agents Chemother..

[34] Sánchez-Aliaga A., Farago P.V., Michél M.D., Sugio C.Y.C., Neppelenbroek K.H., Urban V.M. (2020). Surface Morphology and in Vitro Leachability of Soft Liners Modified by the Incorporation of Antifungals for Denture Stomatitis Treatment. J. Appl. Oral Sci..

[35] Maluf C.V., Janott-Sarlo C.A., Bertolini M.d.M., Menezes L.R., Lourenço E.J.V., Telles D.d.M. (2020). In Vitro Evaluation of Physicochemical Properties of Soft Lining Resins after Incorporation of Chlorhexidine. J. Prosthet. Dent..

[36] Safari S., Barani M., Sadrmohammadi R. (2024). “Antimicrobial Properties of Tissue Conditioner Modified with Chitosan and Green-Synthesized Silver Nanoparticles: A Promising Approach for Preventing Denture Stomatitis”. BMC Oral Health.

[37] Wen J., Jiang F., Yeh C.K., Sun Y. (2016). Controlling Fungal Biofilms with Functional Drug Delivery Denture Biomaterials. Colloids Surf. B Biointerfaces.

[38] Tkaczyk M., Kuśka-Kielbratowska A., Fiegler-Rudol J., Niemczyk W., Mertas A., Skaba D., Wiench R. (2025). The Prevalence and Drug Susceptibility of Candida Species and an Analysis of Risk Factors for Oral Candidiasis—A Retrospective Study. Antibiotics.

