# Supplementary material for: Pterostilbene-Incorporated Tissue Conditioners Exhibit Sustained Antifungal Activity Against Candida albicans In Vitro with Preserved Biocompatibility
Source: Materials (Basel). 2026 May 19;19(10):2126. doi: 10.3390/ma19102126 (PMC13208259; doi:10.3390/ma19102126)
Supplement: Supplementary file 1 [file materials-19-02126-s001.zip › materials-4222349-supplementary.pdf]

Table S1. MEF cell viability after exposure to 24 h eluates from tissue conditioner discs.

Time	TC	TCD	TC-PTE1	TC-PTE2	TC-K
**24 h**	96.08 ± 1.13 (4.6%)	94.44 ± 0.90 (6.2%)	90.67 ± 0.67 (9.9%)	90.31 ± 1.15 (10.3%)	91.82 ± 0.78 (8.8%)
**72 h**	89.77 ± 0.58 (10.8%)	87.87 ± 1.58 (12.7%)	89.69 ± 0.46 (10.9%)	86.19 ± 1.13 (14.4%)	91.36 ± 0.99 (9.2%)
**1 week**	86.01 ± 1.28 (14.6%)	85.09 ± 0.79 (15.5%)	87.72 ± 1.24 (12.9%)	85.63 ± 0.33 (14.9%)	85.37 ± 1.18 (15.2%)

Values are mean ± SEM (n = 3). Numbers in parentheses indicate percent reduction relative to NC. Controls (independent of time): NC: 100.67 ± 0.45 B: 100.70 ± 1.17 10% DMSO: 23.42 ± 0.69 (76.7% reduction) 20% DMSO-1: 14.12 ± 0.44 (86.0% reduction).

Table S2. Within-group time-dependent cytotoxicity comparisons for 24 h exposure.

Group	24 h vs 72 h	24 h vs 1 wk	72 h vs 1 wk
**TC**	**0.004** ** **(6.2%)**	**0.002** ** **(10.0%)**	**0.048** * **(3.8%)**
**TCD**	**0.022** * **(6.5 %)**	**<0.0015** ** **(9.3%)**	0.172 (2.8%)
**TC-PTE1**	0.262 (1.0%)	0.123 (3.0%)	0.222 (2.0%)
**TC-PTE2**	**0.048** * **(4.1%)**	**0.011** * **(4.6%)**	0.659 (0.5%)
**TC-K**	0.711 **(0.4 %)**	**0.009** ** **(6.4%)**	**0.011** * **(6.0%)**

Values show the increase in percent reduction relative to NC between indicated time points. Positive values indicate further decrease in viability. p-values are from unpaired two-tailed t-tests. Asterisks denote significance: * $p < 0.05$, ** $p < 0.01$, *** $p < 0.001$.

Table S3. Between-group cytotoxicity comparisons for 24 h exposure.

**Comparison**	**24 h**	**72 h**	**1 wk**
TC vs TCD	0.262 (1.6%)	0.301 (1.9%)	0.561 (0.9%)
TC vs TC-PTE1	**0.012** * (5.3%)	0.948 (0.1%)	0.431 (-1.7%)
TC vs TC-PTE2	**0.015** * (5.7%)	**0.044** * (3.6%)	0.807 (0.3%)
TC vs TC-K	**0.022** * (4.2%)	0.206 (-1.6%)	0.743 (0.6%)
TCD vs TC-PTE1	**0.028** * (3.7%)	0.298 (-1.8%)	0.170 (-2.6%)
TCD vs TC-PTE2	**0.044** * (4.1%)	0.457 (1.7%)	0.619 (-0.6%)
TCD vs TC-K	0.094 (2.6%)	0.123 (-3.5%)	0.864 (-0.3%)
TC-PTE1 vs TC-PTE2	0.823 (0.4)	**0.028** * (3.5%)	0.121 (2.0%)
TC-PTE1 vs TC-K	0.227 (-1.1%)	0.089 (-1.7%)	0.280 (2.3%)
TC-PTE2 vs TC-K	0.344 (-1.5%)	0.015† (-5.2%)	0.860 (0.3%)

Values are Δ Reduction (%) = (reduction of second-named group) − (reduction of first-named group). Positive Δ indicates that the second group has a higher reduction (lower viability) than the first. Only positive Δ with $p < 0.05$ are marked significant; negative Δ (second group has lower reduction / higher viability) are reported without significance, regardless of p-value. p-values from unpaired two-tailed t-tests; * $p < 0.05$, ** $p < 0.01$, *** $p < 0.001$; ns = not significant ($p \geq 0.05$). Reductions are based on NC = 100.67.

Table S4. MEF cell viability after exposure to 48 h eluates.

Time	TC	TCD	TC-PTE1	TC-PTE2	TC-K
**24 h**	81.00 ± 0.95 (19.5%)	82.42 ± 0.65 (18.1%)	83.11 ± 0.61 (17.4%)	79.22 ± 1.05 (21.3%)	81.57 ± 0.70 (19.0%)
**72 h**	82.79 ± 0.56 (17.8%)	85.51 ± 1.53 (15.1%)	87.70 ± 0.51 (12.9%)	74.99 ± 0.99 (25.5%)	83.93 ± 0.93 (16.6%)
**1 week**	76.23 ± 1.08 (24.3%)	79.16 ± 0.73 (21.4%)	83.35 ± 1.22 (17.2%)	72.20 ± 0.27 (28.3%)	77.43 ± 1.09 (23.1%)

Values are mean ± SEM (n = 3). Numbers in parentheses indicate percent reduction relative to NC. Controls (independent of time): NC: 100.67 ± 0.45; B: 100.70 ± 1.17; 10% DMSO (pooled): 25.71 ± 0.37 (74.5% reduction) 20% DMSO (pooled): 12.56 ± 0.14 (87.5% reduction)

Table S5. Within-group time-dependent cytotoxicity comparisons for 48 h exposure.

Group	24 h vs 72 h	24 h vs 1 wk	72 h vs 1 wk
**TC**	0.204 (-1.77%)	**0.029 * (4.74%)**	**0.012 * (6.51%)**
**TCD**	0.160 (-3.08%)	**0.029 * (3.23%)**	**0.033 * (6.31%)**
**TC-PTE1**	**0.005 ** (-4.56%)**	0.870 (-0.24)	**0.046 * (4.32%)**
**TC-PTE2**	**0.043 * (4.20 %)**	**0.023 * (6.97 %)**	0.110 (2.77%)
**TC-K**	0.120 (-2.35%)	**0.040 * (4.11%)**	**0.011 * (6.46%)**

Values show the increase in percent reduction relative to NC between indicated time points. Positive values indicate further decrease in viability. p-values are from unpaired two-tailed t-tests. Asterisks denote significance: * $p < 0.05$, ** $p < 0.01$, *** $p < 0.001$.

Table S6. Between-group cytotoxicity comparisons for 48 h exposure.

Comparison	24 h	72 h	1 wk
TC vs TCD	0.260 (-1.4%)	0.210 (-2.7%)	0.110 (-2.9%)
TC vs TC-PTE1	0.120 (-2.1%)	0.003 † (-4.9%)	0.012 † (-7.1 %)
TC vs TC-PTE2	0.250 (1.8%)	**0.006 ** (7.7%)**	0.067 (4.0%)
TC vs TC-K	0.650 (-0.5%)	0.370 (-1.2%)	0.480 (-1.2%)
TCD vs TC-PTE1	0.480 (-0.7%)	0.290 (-2.2%)	0.055 (-4.2%)
TCD vs TC-PTE2	0.070 (3.2%)	**0.008 ** (10.4%)**	**0.010 * (6.9%)**
TCD vs TC-K	0.420 (0.9%)	0.440 (-3.5%)	0.260 (1.7%)
TC-PTE1 vs TC-PTE2	**0.045 * (1.6%)**	**0.001 *** (12.6%)**	**0.010 * (11.1%)**
TC-PTE1 vs TC-K	0.170 (-2.3%)	**0.036 * (3.7%)**	**0.022 * (5.9%)**
TC-PTE2 vs TC-K	0.140	0.003 † (8.9%)	0.040 † (-5.2%)

Values are Δ Reduction (%) = (reduction of second-named group) − (reduction of first-named group). Positive Δ indicates that the second group has a higher reduction (lower viability) than the first. Only positive Δ with $p < 0.05$ are marked significant; negative Δ (second group has lower reduction / higher viability) are reported without significance, regardless of p-value. p-values from unpaired two-tailed t-tests; * $p < 0.05$, ** $p < 0.01$, *** $p < 0.001$; ns = not significant ($p \geq 0.05$). Reductions are based on NC = 100.67.
